# Awareness of Sudden Infant Death Syndrome Among Saudi Arabian Women in 2019: A Cross-Sectional Study

**DOI:** 10.7759/cureus.9768

**Published:** 2020-08-15

**Authors:** Razan M Alzahrani, Nada E Algethami, Ali Alharbi, Nasser Alharbi, Rootana Abbas, Lama M Alqthami, Ibrahim Alshardi, Maryam Aljaid

**Affiliations:** 1 Medicine, Ibn Sina National College, Jeddah, SAU; 2 Medicine, Taif University, Taif, SAU; 3 Medicine, Qassim University, Qassim, SAU; 4 Pediatrics, University of Aljouf, Jeddah, SAU; 5 Medicine, Battarji College, Jeddah, SAU; 6 Medicine, Umm Alqura University, Makkah, SAU; 7 Pediatrics, Taif University, Taif, SAU

**Keywords:** sid, sudden infant death, infant, family, medicin

## Abstract

Introduction

Studies on sudden infant death syndrome (SIDS) in Saudi Arabia are limited due to strict religious faith-related observances. The aim of this study was to assess Saudi mothers’ awareness about SIDS.

Methodology

A cross-sectional online electronic survey was administered in different Saudi regions. A self-reported validated Arabic questionnaire was used for collecting data. The study sample included 363 respondents from 384 sample members.

Results

The final sample consisted of 363 (94.8%) respondents, a reduction in size due to 21 non-responders from the total 384 sample members. Two-hundred and thirty-two (63.9%) of the 363 respondents reported not having heard of any SIDS prevention messages, while 36.1% of them had received such messages. Most of the respondents (53.2%) correctly reported that babies should be laid on their backs while putting them to sleep, and only 5.5% reported that babies should be laid in their stomachs when being put to rest. Participants > 50 years, of urban residence, and with primary education had higher knowledge levels. Participants with an age of 18 to 29 years and those with higher education had higher knowledge levels about the correct baby position. Participants with an age between 30 and 39 years, of urban residence, and those with a primary educational level had a higher prevalence of receiving messages about SIDS.

Conclusion

A very good understanding of the proper way to put a baby to sleep among the participants was found, and most of them thought that messages about SIDS were not useful. This study suggests that health education messages regarding SIDS should be directed to all pregnant mothers.

## Introduction

Sudden infant death syndrome (SIDS) is the sudden and unexpected death of an infant less than 12 months old [1]. The cause of death in SIDS cannot be determined by history, examination, or a thorough post-mortem and death scene investigation [1]. The most common causes of infant death are birth defects, preterm birth and low birth weight, maternal pregnancy complications, sudden infant death syndrome, and injuries (e.g. suffocation). SIDS is considered a leading cause of infant death between one and 12 months of age with a male to female ratio of 3:2 [1]. The peak incidence is two to four months with 95% of cases occurring by six months [1]. Most deaths were found to occur between midnight and 8 AM [1]. The cause of SIDS in some infants is multifactorial [2]. Each factor alone is not sufficient to cause death but when expressed in combination with one or more of the other factors will result in death [2]. The triple risk model for SIDS proposed by Filiano and colleagues in 1994 highlights the intrinsic, extrinsic, and additional risk factors for SIDS [3]. Risk factors were divided into modifiable and non-modifiable factors [4]. A previous study in 2017 consisting of 35 interviewed African-American women showed that the mother’s awareness, perceptions, and practices may act to reduce the incidence of SIDS [5]. In the Kingdom of Saudi Arabia (KSA), due to the strict observance of religious faith, studies on SIDS are limited. These limitations are due to lack of granting permission by the Islamic religion for post-mortem tests. This fact limits medical and epidemiological studies on SIDS in Saudi Arabia. The aim of this study was to assess Saudi mothers’ knowledge and attitudes toward SIDS.

## Materials and methods

Ethical clearance for the study was requested, and approval was obtained from Taif University Research Ethics Committee Application No.41-710-0018. A cross-sectional community-based study was done to assess awareness about SIDS among Saudi women in 2019. A sample from different Saudi regions was performed. Participation in the study was done after completing the consent agreement by the participants. Data were collected using an online link from which a self-reporting validated Arabic questionnaire consisting of two sections was obtained. The first section included items for collecting demographic data, and the second section included questions about the participants’ awareness and attitude toward the disease. The desired sample size was determined using the margin of error formula as shown in the equation:

$n=z^2*s^2/e^2$

where *z* = 1.96, the z-score statistic at the 95% confidence interval (CI), and *s^2^ *is the desired variation from the center of the data. Since a 5-point Likert scale was used, s was determined as $s=(max-min)/range=(5-1)/4=1$. *e^2^*, the standard error, was selected at 10%; the minimum value of n was then determined using the equation:

$n=1.96^2*1^2/〖.1〗^2 =384.16$

However, given that the study was not an inference design study, more sample participants were allowed into the study. The final sample consisted of 363 respondents.

Data analysis was carried out using the Statistical Package for Social Sciences (SPSS) version 25 (IBM Corp., Armonk, NY, USA). Qualitative data were expressed as number and percentage, and the chi-squared test was used to assess the relationship among variables. A p-value < 0.05 was considered significant.

## Results

About 63% of the respondents (63.9%) reported no previous knowledge about SIDS prevention messages. Most of them had an urban residence (76.9%), 64.2% had an age ranging from 18 to 29 years, and 76% had higher education levels (Table 1).

**Table 1:** Demographic variables frequencies SIDS: sudden infant death syndrome

Variable	Freq. (%)	
Age	18-29	233(64.2)
	30-39	83(22.9)
	40-49	39(10.7)
	50>	8(2.2)
Residence	Rural	84(23.1)
	Urban	279(76.9)
Number of children	None	213(58.7)
	1	42(11.6)
	2-3	52(14.3)
	>3	56(15.4)
	Primary school	3(0.8)
	Preparatory school	19(5.3)
	Secondary school	65(17.9)
	Higher education	276(76.0)
Heard of SDIS	Yes	131(36.1)
	No	232(63.9)

A significant relationship was found between participants’ knowledge about SIDS and their ages, residences, and educational levels. In KSA, educational levels before the university stage include a primary school, preparatory (intermediate or middle school), and secondary school. Those individuals > 50 years with rural residence and those with primary education levels had higher SIDS knowledge (p ≤ 0.05), as shown in Table 2.

**Table 2:** Associations between perceived knowledge of SIDS and demographic variables A: Test of association B: Test of differences between groups SIDS: sudden infant death syndrome

Variable		Freq. (%) Agree	p-value
Knowledge of SIDS			
Age	18-29	76(32.6)	0.103^A^ 0.000^B^
	30-39	31(37.3)	
	40-49	19(48.7)	
	50>	5(62.5)	
Residence	Rural	32(38.1)	0.662^A^ 0.000^B^
	Urban	99(35.5)	
Number of children	None	70(32.9)	0.382^A^ 0.052^B^
	1	15(35.7)	
	2-3	23(44.2)	
	>3	23(41.1)	
Education	Primary school	3(100)	0.372^A^ 0 .000^B^
	Preparatory school	9(50)	
	Secondary school	20(30.8)	
	Higher education	99(35.9)	

**Figure 1:**
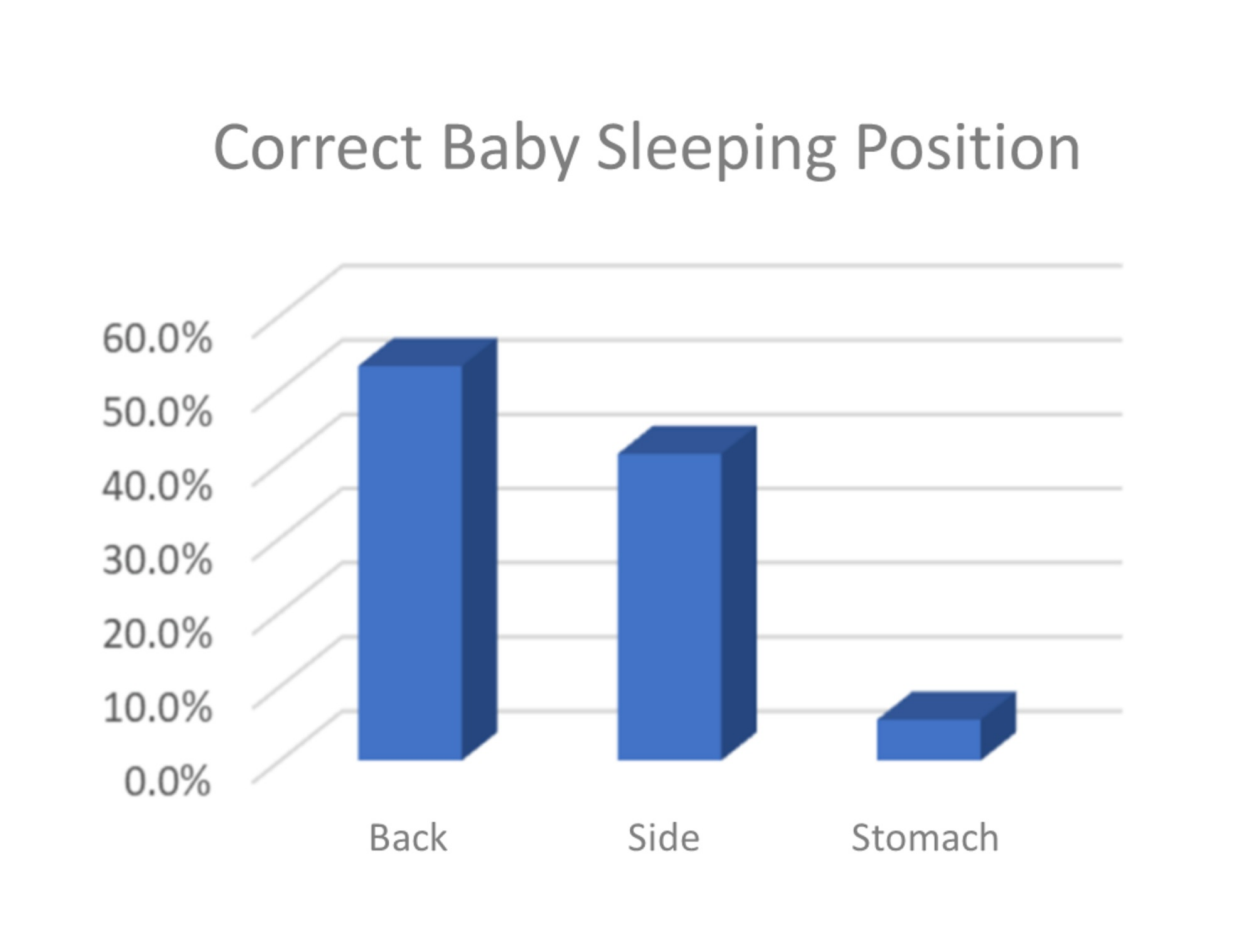
Frequency distribution of correct baby sleeping position

Over half of the respondents (53.2%) correctly reported that babies should be laid on their backs while putting them to sleep, while only 5.5% of them reported that babies should be laid in their stomachs when being put to rest (Figure 1).

A significant relationship was found between knowledge about the correct baby position and participants’ ages and educational levels. Participants aged 18 to 29 years and those with higher education levels had the highest knowledge levels (p ≤ 0.05), as shown in Table 3.

**Table 3:** Correct baby sleeping position and demographic variables A: Test of association B: Test of differences between groups SIDS: sudden infant death syndrome

Correct sleeping position for baby		% correct	p-value
Age	18-29	134(57.5)	0.425^A^ 0.000^B^
	30-39	38(45.8)	
	40-49	17(43.6)	
	50>	4(50.0)	
Residence	Rural	48(57.1)	0.319^A^ 0.264^B^
	Urban	145(52)	
Number of children	None	124(58.2)	0.169^A^ 0.048^B^
	1	21(50.0)	
	2-3	23(44.2)	
	>3	25(44.6)	
Education level	Primary school	0(0.0)	0.004^A^ 0.000^B^
	Preparatory school	2(11.1)	
	Secondary school	32(49.2)	
	Higher education	158(57.2)	

**Figure 2:**
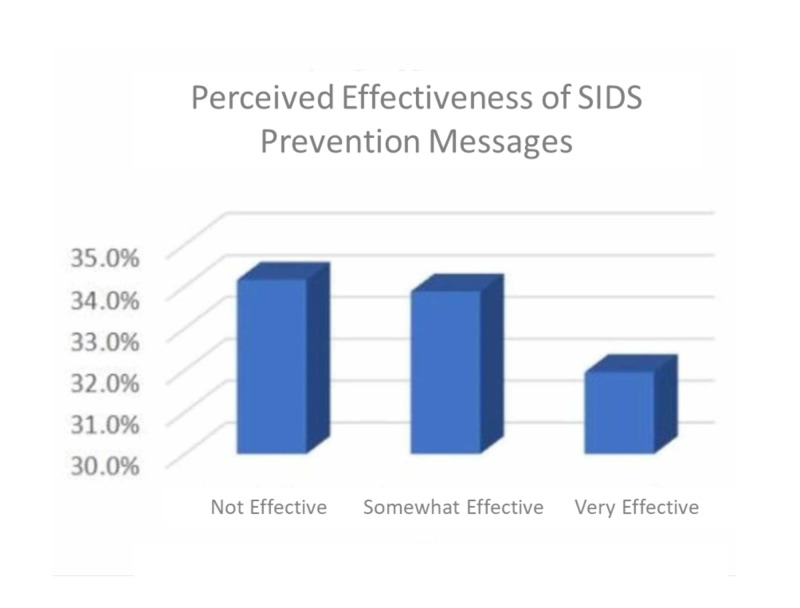
Frequency distribution effectiveness of SIDS messages SIDS: sudden infant death syndrome

More than one-third (34.2%) of the respondents felt that messages regarding SIDS were not at all effective, and 33.9% of them felt that these messages were somewhat effective (Figure 2). 

Table 4 shows that a significant relationship was found between the participants’ ages, residences, and educational levels and having received messages about SIDS. Those aged 30 to 39 years, those of urban residence, and those with a primary educational level had a higher prevalence of those reached by this message (p ≤ 0.05).

**Table 4:** Messages against SIDS across demographic variables A: Test of association B: Test of differences between groups SIDS: sudden infant death syndrome

Messages about SID		% Yes	p-value
Age	18-29	13(5.6)	0.290^A^ 0.000^B^
	30-39	9(10.8)	
	40-49	4(10.3)	
	50>	0(0.0)	
Residence	Rural	3(3.6)	0.145^A^ 0.007^B^
	Urban	23(8.2)	
Number of children	None	14(6.6)	0.641^A^ 0.058^B^
	1	5(11.9)	
	2-3	3(5.8)	
	>3	4(7.1)	
Education level	Primary school	1(33.3)	0.045^A^ 0.002^B^
	Preparatory school	2(11.1)	
	Secondary school	9(13.8)	
	Higher education	14(5.1)	

**Figure 3:**
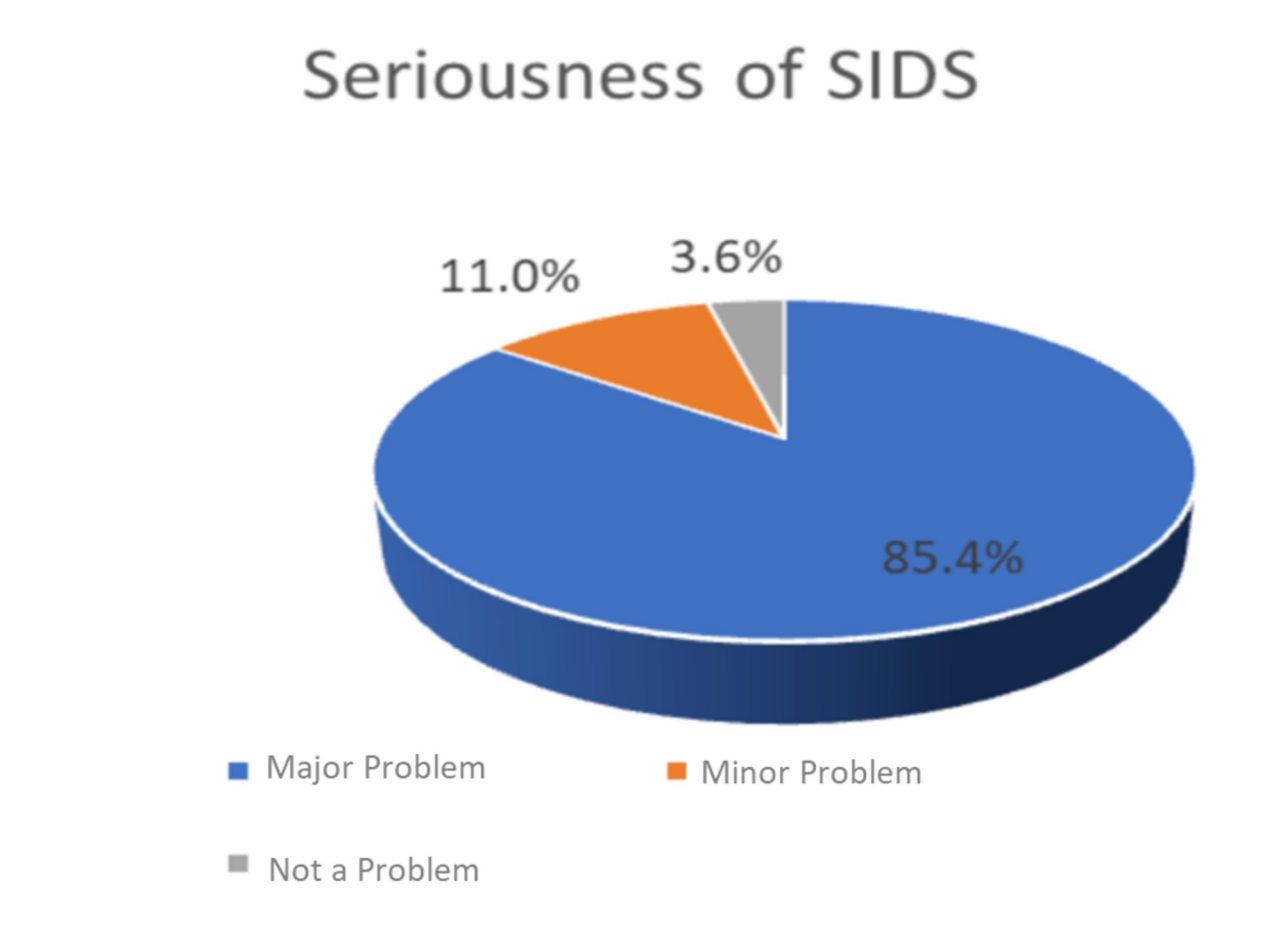
Distribution of responses about seriousness of SIDS SIDS: sudden infant death syndrome

More than 85.4% of the respondents believe that SIDS is a major problem, and 3.6% believe that SIDS is not a serious problem (Figure 3).

A significant relationship was found between the participants’ ages, number of children, and educational levels and whether they thought that SIDS is a serious problem. Individuals aged 18 to 29 years, not having children, and having a secondary level of education were a higher percentage of the participants who thought that the disease is a serious problem (p ≤ 0.05), as shown in Table 5.

**Table 5:** Seriousness of SIDS across demographic variables A: Test of association B: Test of differences between groups SIDS: sudden infant death syndrome

Seriousness of SIDS		Major problem (%)	p-value
Age	18-29	203(87.1)	0.104^A^ 0.000^B^
	30-39	70(84.3)	
	40-49	32(82.1)	
	50>	5(62.5)	
Residence	Rural	71(84.5)	0.378^A^ 0.414^B^
	Urban	310(85.7)	
Number of children	None	187(87.8)	0.641^A^ 0.048^B^
	1	34(81.0)	
	2-3	44(84.6)	
	>3	45(80.4)	
Education level	Primary school	2(66.7)	0.111^A^0.010^B^
	Preparatory school	15(83.3)	
	Secondary school	56(86.2)	
	Higher education	236(85.5)	

## Discussion

The present study aimed to understand the knowledge and attitude toward SIDS among Saudi women. The required sample for the present study was 384 from the computation of the sample size for the study. Due to incomplete answering of the questions, some observations were deleted, leading to a reduction in the sample size to a statistically large enough size of 363 [6]. 

In the present study, knowledge about SIDS and a participant’s residence were statistically related since participants from rural regions had more knowledge than those from urban regions. This result disagrees with previous studies in which those from urban areas had better knowledge [7]. These studies found that people in urban areas would be more knowledgeable due to having many different sources of information about SIDS. In addition, better infrastructure enabling access to information could be the cause [7]. This difference could be also due to differences in traditional knowledge of taking care of infants among rural area dwellers compared to those from urban areas [7].

Knowledge also seemed to be determined by the number of children one has, since larger proportions of participants with at least one child had better knowledge than those without any children. The reason could be that medical staff inform parents about SIDS upon the birth of their first child [8]. Participants with lower levels of education formed a higher percentage of those who had better knowledge about SIDS. This finding could be explained by their relatively lower percentage of the study population in this study.

A good proportion of the respondents (85%) knew the correct position to put a baby to sleep, which is lying the child on his/her back. This impressive number could be explained by the effects of both traditional and modern forms of medical knowledge that are used to explain ways of safely handling infants to new parents, especially mothers [9]. Additionally, parents with more than one child were found to inform new parents about safe ways to handle babies and infants [8]. It was also found that individuals with higher education levels consisted of a higher percentage of those who knew the correct position to place a baby to sleep, a finding that was consistent with those of other studies [8,10].

About 85% of the participants (85.4%) mentioned that SIDS is a major problem. This percentage tended to decrease across the age groups. This finding could be attributed to knowledge of some methods for SIDS prevention. These methods include the proper positioning of placing the baby to sleep and knowledge that infants and parents should not sleep in the same bed due to the risk of adults lying on top of the infants and suffocating them. The same result was observed in the section about self-reported number of children and depended on the number of children a participant had. These findings were in agreement with those of other studies [8].

## Conclusions

Our findings showed that most participants had a very good understanding of the proper way to put a baby to sleep. Most of the respondents reported that they understood the seriousness of SIDS but thought that messages about SIDS were not useful. A small percentage of the participants reported that they did not receive SIDS messages. This study suggests that health education messaging regarding SIDS should be directed to all pregnant mothers. By improving understanding of SIDS risk reduction practices among mothers, SIDS-related deaths can be prevented.

## References

[1] (2019). Investigation of Sudden Infant Death Syndrome (Diagnostic Pediatric Pathology).

[2] Duncan JR, Byard RW (2018). Sudden infant death syndrome: an overview. SIDS Sudden Infant and Early Childhood Death: The Past, the Present and the Future.

[3] Filiano JJ, Kinney HC (1994). A perspective on neuropathologic findings in victims of the sudden infant death syndrome: the triple-risk model. Biol Neonate.

[4] Konstat-Korzenny E, Cohen-Welch A, Fonseca-Portill R, Morgenstern-Kaplan D (2019). Sudden unexpected infant death: review and analysis of adherence to recommendations. Cureus.

[5] Montgomery F (2014). Sudden Infant Death Syndrome Awareness in Relation to the Perceptions of African American Women. National Medical Fellowships.

[6] Burmeister E, Aitken LM (2012). Sample size: how many is enough?. Aust Crit Care.

[7] De Vaus D (2001). Research Design in Social Research, First Edition. First Edition.

[8] Nofal HK, Abdulmohsen MF, Khamis AH (2011). Incidence and causes of sudden death in a university hospital in eastern Saudi Arabia. East Mediterr Health J.

[9] Hoffman HJ, Hillman LS (1992). Epidemiology of the sudden infant death syndrome: maternal, neonatal, and postneonatal risk factors. Clin Perinatol.

[10] Hauck FR, Thompson JM, Tanabe KO, Moon RY, Vennemann MM (2011). Breastfeeding and reduced risk of sudden infant death syndrome: a meta-analysis. Pediatrics.

